# BSVA: blockchain-enabled secured vertical aggregation algorithm for transactions management in drug traceability framework

**DOI:** 10.1038/s41598-025-12641-z

**Published:** 2025-07-31

**Authors:** P. Bhuvaneshwari, Y. Harold Robinson, M. Bagya Lakshmi

**Affiliations:** 1 Manipal Institute of Technology Bengaluru, Manipal Academy of Higher Education, Manipal, India; 2 Department of Computer Science and Engineering, Francis Xavier Engineering College, Tirunelveli, India

**Keywords:** Drug traceability system, Secured vertical aggregation algorithm, Certificate authority, Blockchain technology, Supply chain management, Drug regulation, Drug discovery

## Abstract

The pharmaceutical supply chain has a critical component, the Drug Traceability System, which tracks drugs from manufacturers for further processing and distribution. The integration of blockchain technology yields a secure solution for monitoring drugs throughout the supply chain management process. The paper proposes a novel Blockchain-enabled Secured Vertical Aggregation Algorithm (BSVA) by leveraging the Hyperledger model. The proposed model minimizes the requirement for a centralized authority to ensure privacy while also enhancing scalability to reduce response time in the process of managing transactions on the Blockchain. The Certificate Authority is used to maintain a secure data-sharing process. The robust aggregation is used for the local models to process the chain code, ensuring the successful execution of the secured transaction. The smart contract is deployed into a blockchain model as the block is stored and linked to the distributed Ledger. The decentralized framework is used by chain code, which guarantees that transactions are highly transparency. The performance parameters demonstrate the efficiency of the proposed model by enhancing the overall performance of the drug traceability system, as the proposed algorithm ensures the integrity of pharmaceutical products throughout the supply chain.

## Introduction

Drug traceability is the process of identifying the origin of drugs through stakeholders in the supply chain management to track transactions executed within the system^1^. The development of a successful traceability framework is a significant component of regulatory authorities worldwide, enabling the tracking of the traceability of specified drugs. The healthcare industry^2^ deficiency in the implementation of the framework has the solution to the drug traceability-related problem, including the secured network to analyze the authentication process into the stakeholders of the supply chain management. The tracking and authentication of drugs within the transportation into the distribution networks need a specific electronic system as several techniques have been implemented like mobile environment-enabled solutions, Machine learning computational solutions, serialization process, RFID tags, bar codes with serial numbers for the verification process of the drugs into the specific views^3^. The drug-based tracking system^4^ is involved in maintaining transactions in the pharmaceutical industry through the drug transaction process, which consists of providing authentication for product purchase management systems. Moreover, the solutions haven’t been interoperable and didn’t produce a particular influence on avoiding fake drugs to protect the lives of the patients.

The blockchain framework provides a solution for supply chain management systems, resulting in significant advancements and an increasingly transparent approach to managing transactions among stakeholders^5^. Blockchain combines every possible solution to create a distributed data management system that supports drug-related actions, enabling the identification of fake drugs through a transparent and accountable process^6^. The blockchain-enabled solution minimizes the use of trusted centralized techniques, resulting in a decentralized solution for various pharmaceutical management applications. It helps reduce impairments in the supply chain to facilitate collaboration among commonly untrusted stakeholders, thereby enabling the decentralized traceability framework^7^. Blockchain confirms that the drugs have been delivered to an authorized stakeholder by making a validated Medledger^8^ of the entire transaction available in the system. Third-party service providers cannot be directly involved in the digital transfer process, as the blockchain-based solution is used to provide robust management for identifying fake drugs by utilizing the information gathered during transaction management to record it securely in the shared Ledger^9^.

Blockchain-based supply chain management has provided reliability by establishing trust among stakeholders, replacing third-party service providers with tracking functionality that enables the transparent sharing of transaction details, thereby facilitating the implementation of the process. This is because every stakeholder in the traceability system must be able to track the products^10^. The primary motivation within the system is to facilitate the workflow among multiple stakeholders through the Hyperledger model, which enables the deployment of a permissioned network. This is achieved by enrolling trusted stakeholders into a specific service. The chain-code^11^ within the supply chain enables stakeholders to share transaction data, empowering them to retrieve sensitive information from multiple integrated stakeholders in the supply chain management system, thereby building confidence among industry stakeholders. Cryptography utilizes connections within number theory to construct higher security than conventional public key encryption functionality while also employing authentication-based access control processes^12^.

The drug traceability system has the potential to transform the pharmaceutical industry by providing a secure and efficient way to track products throughout the supply chain. The proposed model is designed to provide benefits such as enhanced security, efficiency, and transparency. The utilization of the blockchain chain model guarantees that entire transactions are tamper and transparent, minimizing counterfeiting and ensuring the safety of patients. The decentralized framework enables successful transactions, making reliable and resilient communication possible. The pharmaceutical products have authenticity and integrity, which enhance their performance, as the executor has confirmed that the chain code is executed securely.

The proposed BSVA model has been constructed to provide a secure and scalable solution for data sharing with enhanced traceability. The proposed algorithm offers an improved contribution to pharmaceutical supply chain management. The Blockchain technique is incorporated to ensure the integrity of transactions by eliminating the need for a centralised authority. The transparent tracking process is facilitated efficiently through the authentication procedure. The robust design of the proposed framework has yielded improper performance in metric transactions, such as assessing time. The proposed model is designed to address issues related to large-scale deployment in the pharmaceutical industry. Additionally, the proposed BSVA algorithm ensures the integrity of pharmaceutical products throughout the supply chain, thereby minimizing counterfeiting and improving efficiency.

The main contribution of the paper is:

- The proposed Blockchain-enabled Secured Vertical Aggregation Algorithm (BSVA) enables secured data sharing in the pharmaceutical supply chain by leveraging blockchain functionality to ensure secured transactions.
- The proposed framework eliminates the need for a centralised authority by enabling a decentralised architecture, thereby increasing the scalability of the drug traceability system.
- The proposed BSVA algorithm ensures the integrity of pharmaceutical products in the supply chain through a robust solution to complete the tracking process.
- The proposed Blockchain-based model guarantees security by leveraging smart contracts to minimize risks.
- The chain code authorizes transactions to be validated, minimizing the risk of disputes and errors.
- The proposed algorithm demonstrates improved performance metrics, including a minimised transaction process time and increased transactions per second, making it well-suited for large-scale deployment.

## Related works

The pharmaceutical supply chain comprises several stakeholders, including manufacturers, authorities, distributors, and patients, who are all involved in the supply chain process. The complexity of these transactions necessitates an efficient system for addressing the present issues^13^. The traceability process must prioritize regulatory matters to ensure the security of the particular product. In contrast, blockchain-enabled traceability offers a solution for managing a distributed platform as a transparent system^14^. Hyperledger Besu^15^ addresses the crucial needs for drug traceability, including security, authorization, and transparency, while also providing scalability, which helps solve the challenges faced by the pharmaceutical industry. The Ethereum technique^16^ is implemented to combine with smart contracts, enabling the decentralization of off-chain storage and producing effective traceability in the healthcare system. This is achieved by making the data origin transparent, thereby reducing the need for intermediate providers to secure transactions for every stakeholder.

A decentralized Hyperledger framework^17^ features a confidentiality process that enables off-chain storage for rapid transactions through smart contracts, facilitating data transmission by enhancing security through data encryption. The Blockchain-based Traceability of Counterfeited Drugs (BBTCD) technique^18^ has been developed to track counterfeit drugs through the Ethereum blockchain, providing transparency at an effective cost. The SecureMeds^19^ methodology has been implemented to prevent drug counterfeiting throughout the supply chain system, which has led to major disasters. Meanwhile, the decentralization technique has ensured transparency in the medicine supply chain, thereby overcoming security-related traceability issues. Every product has authenticated certificates that are verified through the authority of the drug management to enhance the product’s reliability. Non-fungible tokens (NFTs)^20^ have been developed with the blockchain concept like smart contracts for improving integrity and traceability in supply chain management. NFT transferability has been examined to enhance the efficiency and reliability of the system by providing solutions to the specific issues addressed in supply chain management.

Blockchain-enabled drug traceability^21^ provides a solution to enhance several key drug traceability functions, ensuring specific identification in the pharmaceutical industry and maintaining supply chain integrity to address complex issues. Blockchain-based IoT^22^ has been involved in mitigating the extensive losses incurred by the pharmaceutical industry by enhancing the verification process through medicine authentication, which enables the monitoring of ineffective medicines. This improvement enhances security, traceability, and visibility in supply chain management. The blockchain framework^23^ utilizes a distributed Ledger to strengthen authentication and transparency in traceability, thereby increasing transparency and trust in multi-chain integration. A game-theoretic approach^24^ has been proposed as a solution for achieving drug traceability, thereby addressing the trade-off complexities associated with sensitive drug consumption. A fully decentralized blockchain-enabled framework, NFT-IoT Pharma Chain^25^, has been integrated with IoT devices into the chain code to enhance traceability by providing data integrity through blockchain non-fungible tokens, thereby securely implementing the validation process.

.

The PHTrack technique^4^ has been implemented to overcome scalability problems in drug supply chain management, providing a developed approach that integrates the procedure with secure off-chain transmission to ensure reliable drug supply chain traceability functionality. The cyberattacks have been identified through the combined Use of Blockchain with Machine Learning functionality^26^ to demonstrate several key characteristics, including security, integrity, and accountability, in drug chain management. The regulatory authority must issue certificates based on the ownership contribution recorded in the blockchain ledger, which stores metadata in off-chain storage using chain-code to establish end-to-end security.

BDLT-IoMT technique^27^ has been implemented to provide a robust data process using a blockchain model for ensuring the communication within nodes to resolve the issues of resource management and scalability. The BAIoT-EMS method^28^ has been designed to enhance security in Enterprise Management Systems by utilizing smart contracts to optimize device management using available resources. The blockchain framework^29^ has been constructed to provide enhanced energy management by ensuring data privacy. The decentralized, adaptive blockchain framework^30^ has been used to develop intelligent transport systems for addressing real-time monitoring issues. The successful distributed transaction has been executed through the Blockchain-enabled multithreading functionality^31^ to ensure lightweight consensus methodology for private blockchain network. Advanced Practical Byzantine Fault Tolerance (APBFT) Algorithm has been implemented for ensuring scalability in Internet of Medical Things by enabling efficient integration of several healthcare stakeholders. The hybrid cryptographic technique of Threshold Proxy Re-Encryption has been used to enhance the security in IoMT^32^.

While several blockchain-enabled techniques have been involved for drug traceability system, the Hyperledger model could need substantial computational resources. Ethereum has the scalability issue according to the consensus functionality to reduce the transaction processing time. Decentralized Hyperledger functionality has the complexity for implementing the transaction and need substantial expertise in blockchain model. The Counterfeited Drugs traceability model could need substantial modifications in the supply chain model that could be time-consuming. Non-fungible tokens could be exposed to counterfeit and the utilization in drug traceability systems. Moreover, the architectures having the utilization of blockchain model in pharmaceutical supply chain could produce uncertainty and challenges.

## Proposed work

The proposed technique features an enhanced traceability framework that is utilised to improve the pharmaceutical supply chain management system, facilitating the tracing and validation of transactions. The Hyperledger framework with the Blockchain technique utilizes the need for a common authority for providing privacy, efficiency, and scalability through the traceability of information and generally minimizing the response time for the storage and sharing of the transaction in the blockchain framework. The trusted atmosphere is created within the users, the Hyperledger supplies the uniqueness management process which utilizes the user and improves the authentication process for every user in the network. It maintains the membership process which integrates the rules by several supply chain management people to produce the validation, authentication and verification. The Access control could be utilised for providing the extra process of permission as the particular user Id must be permitted to process the Chain code framework but not permitted to deploy it. The membership process is the innovative framework which process resource management, minimizing the attack in the participated users in the health care management process.

The complexity of Pharmaceutical Supply Chain has produced the significant challenges to ensure the integrity of drugs. The improved efficient transactions management into the drug traceability framework as the transparency related issues is the critical aspect of these challenges to make it hard to verify the drug movement from the manufactures into the users. This kind of problem will lead to the proliferation of counterfeit drugs to create major health risks to consumers and distrust in the pharmaceutical industry. Additionally, the capability of discovering counterfeit products to ensure public health and safety. The lack of interoperable systems in supply chain management could make it hard to achieve seamless traceability. To address the issues, the requirement for an integrated transaction management framework that can effectively track drug transactions throughout the entire supply chain, the proposed model should incorporate a robust blockchain model to ensure public health. The current supply chain models lack the transparency required for efficient tracking of drugs throughout the supply chain. The accuracy of the secured transaction data is essential for preventing errors. The interoperability of several stakeholders is critical for providing an efficient traceability process. The proposed BSVA algorithm is used for providing the enhanced performance in the Hyperledger framework to implement traceability in the pharmaceutical supply chain. The BSVA algorithm leverages the Hyperledger framework for creating a secured system to manage transactions and tracking products in the supply chain. The utilization of blockchain technology provides the integrity of the data as the Vertical Aggregation methodology provides the secured data sharing within the stakeholders. The BSVA algorithm illustrates a unique model for enhancing the transaction and traceability in the pharmaceutical supply chain. The utilisation of blockchain-enabled vertical aggregation demonstrates a secure and transparent solution to minimise counterfeit product risks, thereby providing greater patient safety.

**Fig. 1:**
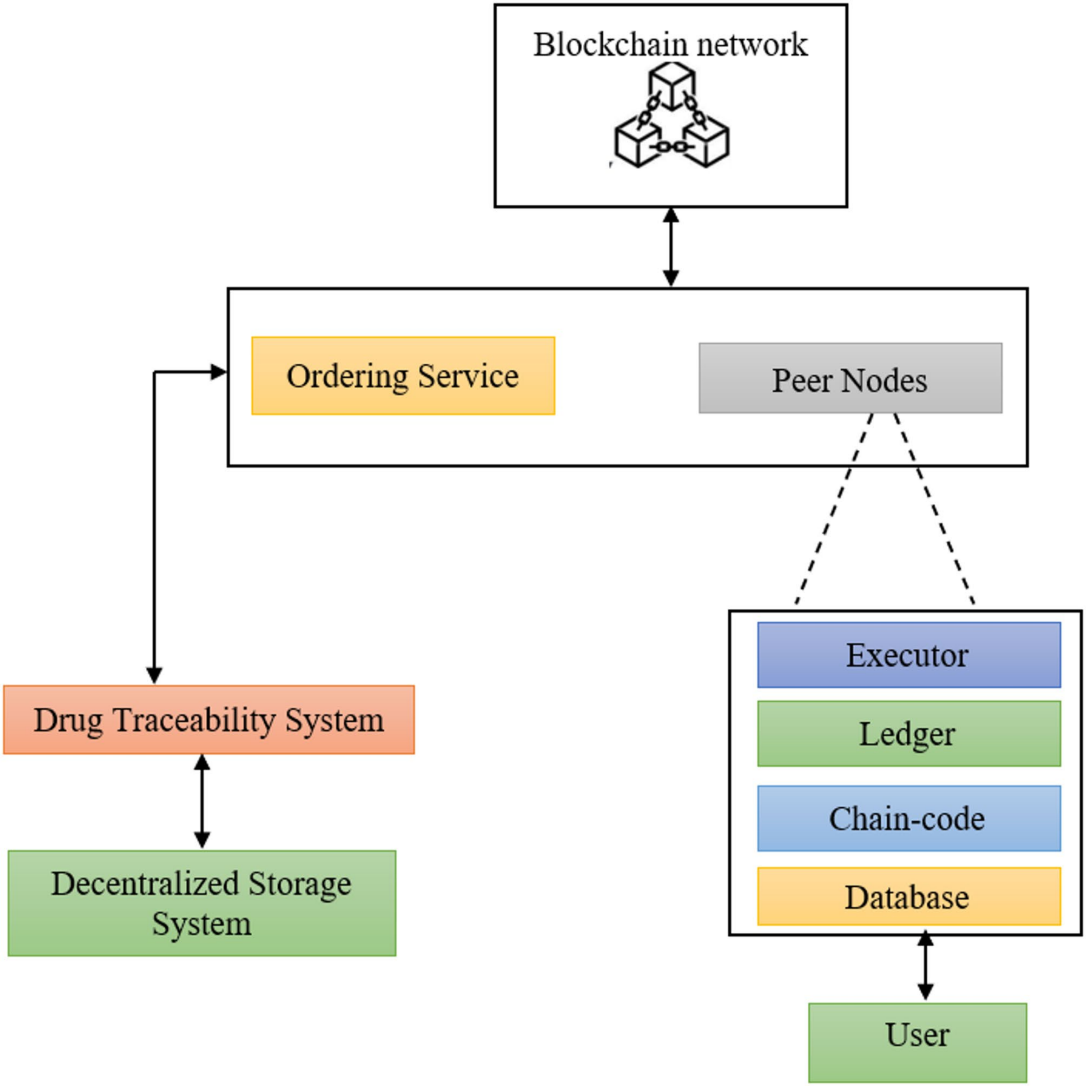
Drug traceability system.

The Drug Traceability system is a blockchain-based model that confirms the integrity of pharmaceutical products throughout the supply chain. The system saves data in a decentralized storage to maintain through a network node for validating the transactions. The ordering service is used to order transactions by building blocks and validates them through peer nodes. After validation, they will be included in the Blockchain. The peer nodes are connected to the user to maintain the ledger with details about the transaction history. The executor functionality demonstrates the chain-code that ensures the secured execution model. The Ledger affords a transaction of tamper-proof record as the validated transactions are recorded in a reliable way and it is illustrated in Fig. 1.

The Hyperledger framework is designed to manage the configuration of resources using previously defined procedures. The information has been stored in several ways; the consensus technique could be utilized to enhance the security features of healthcare record maintenance.

### Certificate authority

The proposed technique has been used to manage the private blockchain framework of several untrusted used assets in the supply chain management system. Users are involved through the validation of certificates, and the authentication process is used to provide the highest confidentiality and privacy. Every participating user is involved in a common root certificate which binds particular components within the organization through assigning the specific Certificate Authority, a distinctive network while the user can utilise the Authority as the communication within the Hyperledger framework could be generated through the private key and public key of Certificate authority, they are having the responsibility for the revocation and the renewal of several certification types issued into the users of the particular organization.

### Peer

It could be the Blockchain framework channels that update the ledger, which contains the Transaction log, while the various peers in the blockchain coordinate with each other inevitably. A peer can utilise the commit node as part of the transaction process, which is delivered to the network to produce the updated policies.

### Executer

The service utilizes the transaction process which is endorsed by the Peers on the Blockchain blocks. The transaction has the signatures of each node which are delivered to the service while the service is committed into the Ledger. It communicates the blocks to the peers on the blockchain framework for validating the consensus in the endorsement process. The executor determines the consensus within the transaction process while the blocks have been validated and committed into the Ledger using the services of the Ledger.

**Fig. 2:**
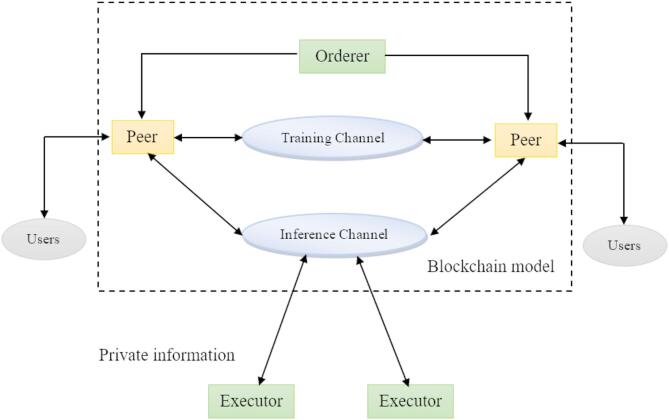
Blockchain-based model.

The capability to protect the private data of stakeholders is the primary feature of the proposed model. Stakeholders can connect to the blockchain through applications that provide a secure interface for interacting within the network. The model ensures that sensitive data is encrypted to protect it from unauthorised access, allowing stakeholders to participate in the blockchain network and benefit from its transparency features. Figure 2 illustrates the proposed framework, which includes the Orderer, peers, and executors for the users, while the third party maintains private information that connects to the blockchain through the applications. Multiple channels are involved in providing the prediction to the inference channel for security purposes. The smart contracts have data collection capabilities that facilitate the transaction flow for the aggregation process in a secure manner.

The proposed technique’s improvement incorporates a traceability framework that enhances supply chain traceability and validates transactions. The Hyperledger blockchain technique necessitates an authority to achieve enhanced privacy and scalability through access control, specifically minimising response times for transactions within the blockchain network. The trusted framework is segregated from the untrusted participants, while Hyperledger is used to manage authentication services for every participant in the network. It involves the membership process, which maintains the regulations of the supply chain management. Several supply chain management providers are involved in managing the resource utilisation process with an improved authentication process. The membership process is an innovation design that manages the entire healthcare management process. The list of Access controls is used to provide an extra permission procedure, in which the particular user ID is permitted to invoke the blockchain technique before deployment.

**Fig. 3:**
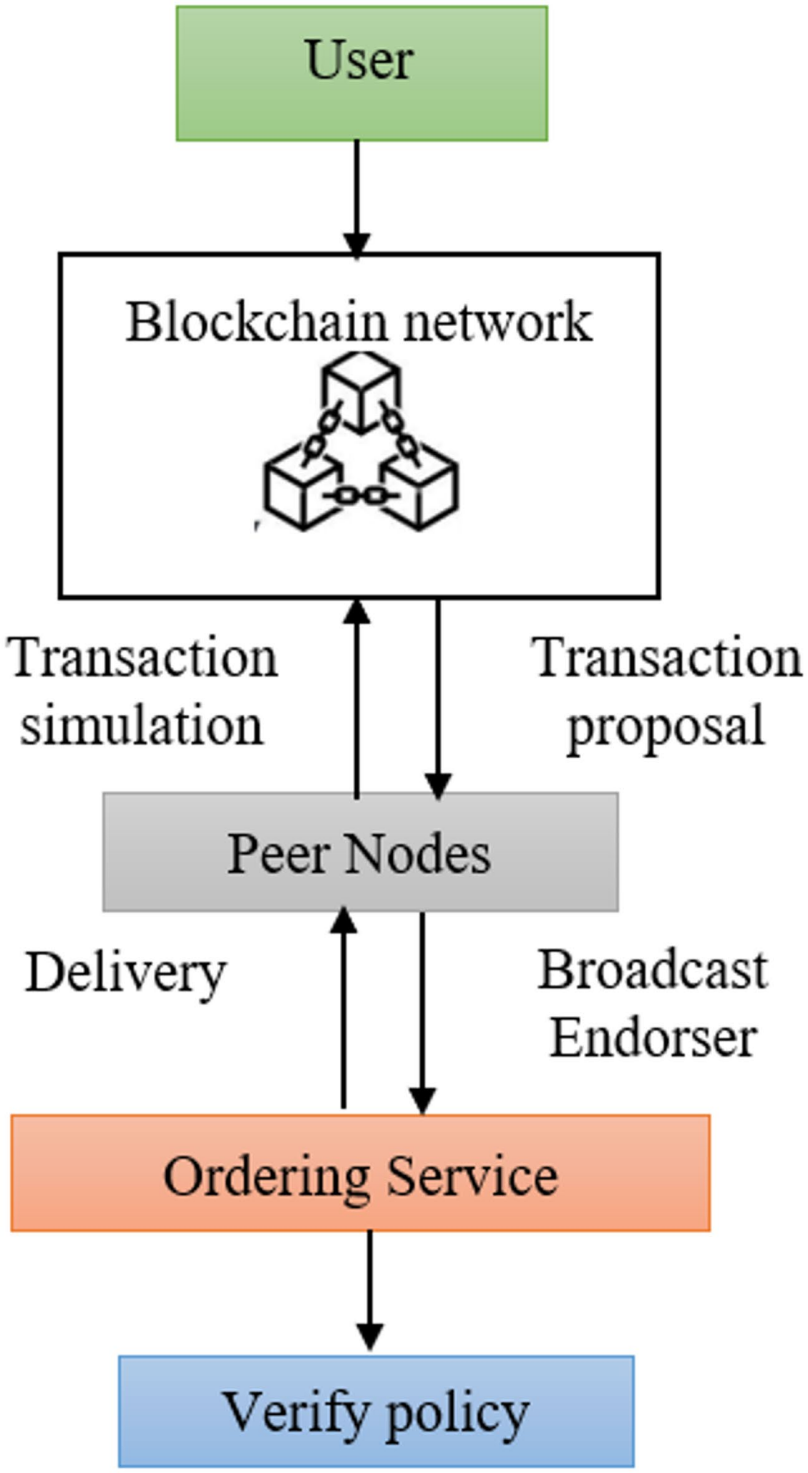
Transaction model.

The transaction model in the blockchain network is constructed through the complex parameters with different components. The request from the user is delivered through the peer nodes to simulate the transaction and validity verification process. The peer nodes utilise a policy for demonstrating that a valid transaction is to be committed to the Blockchain. The ordering service is responsible for processing transactions in a specific sequence, ensuring a successful transaction. The validation process is completed through the blocks whenever the transaction is completed. The policy verification process ensures the validity of blockchain transactions in the network. The blockchain state is verified to reflect the latest transactions by ensuring that every node in the network maintains a consistent view of the blockchain model. The transaction model offers several benefits, including consistency, transparency, and security, through the decentralised network, as illustrated in Fig. 3.

The network manager manages access control to maintain the Hyperledger, which enables the configuration of resource utilisation through provided policy management. The shared data is stored in several ways; the consensus technique can provide secure transmission using consensus management to enable safe and reliable transmission within a set of untrusted managers. The permission to enter the Blockchain network is demonstrated through consensus among participants, while the framework utilises consensus management to enable and execute ledger transactions. The effectiveness of the framework is compared with the public Blockchain, as it processes 3,550 transactions in one second. The standard features of the ledger technique are:

1. It provides private permission and an enhanced framework for executing multiple transactions within the Blockchain framework.
2. The flexibility model leverages consensus among users within the network.
3. It involves an improved framework that utilises transaction integrity among the channels.
4. It permits the creation of channels within the segregate member management for transmitting and accomplishing the privacy.
5. It enables specific improvements in Blockchain coding.
6. The processing of transactions is minimal in terms of latency, compared to related Blockchain frameworks.
7. It has several query types, including range queries and chain queries.

The development and distribution procedure for delivering products has been identified as a complex process, as the traceability from origin within manufacturers and the provision of services to large-scale healthcare systems could be enhanced using the blockchain technique to create a traceability system for sourcing data, delivery processes, and manufacturer information. The proposed system should ensure that log details regarding the storage system, from chain coding to tracking, are maintained for every transaction executed by stakeholders at different levels, thereby facilitating the supply chain process. The supply chain process demonstrates several stakeholders.

1. Supplier: The supplier is the primary stakeholder in the supply chain process, providing materials to another manufacturer. The details of the supply chain system are recorded, including the name, code, supplied quantity, date, and other related information so that stakeholders can identify the material source.
2. Manufacturer: The purchase of materials from suppliers is facilitated through traceability, and this process is integrated into the manufacturing of components to produce the expected values. These developed materials can then be distributed to stakeholders. The owner of the traceability system is accountable for the encoding procedure through the monitoring experts. Whenever the encoding procedure is completed, the manufacturer delivers the products into the blockchain ledger where every packaged unit must consist of the product code to enable the dynamic tracking process within the supply chain management. The manufacturer has the accountability for monitoring and maintaining the product-related information on drugs and stores the relevant data in a decentralized file management system with a high level of integrity.
3. Wholesale distributor: The responsibility for ensuring effective and reliable transactions with stakeholders in the supply chain management system includes various services, such as distribution, packaging, and order fulfilment.
4. Retailers: The purchase of materials from wholesale distributors has been delivered to users through the traceability system, which categorises criteria and distributes services to consumers.
5. Governing authority: The Authority has a specific role in maintaining the quality and safety of distributing and storing basic information that manages products utilising storage management and maintains the products for delivery to the market.

The traceability has the functionality of categorising the backwards process of trace, while the dynamic supplier and the user develop transaction-related data. The position of the materials utilises the forwarding process, as the exact position of the supply chain-related data within the transaction flow has some limitations for the end-user. The chain code is a mutual agreement between the active nodes in the network, as the deployment framework determines the participation of stakeholders in the connected channels, enabling the flow of transactions into the cyber parameters of the entire network. The transaction flow is categorised into the physical flow of relevant data through the entire supply chain framework, from one user to the remaining users. The chain code has been deployed within stakeholders, ensuring the integrity and quality of the process and allowing them to monitor transaction records at any time.

The stakeholders of the supply chain management have been initialised and regulated through the authority of a communication-based blockchain network. The registration process is implemented through the chain code, as the regulation authority manages the network. The permission-enabled network creates an additional security layer to communicate with the system using the virtual private network. The data includes users’ addresses, contact details, and related information regarding stakeholders and suppliers. The authority will verify the data and maintain the chain-code data as the process finishes, and the stakeholders complete the process in the network. The register process involves transactions that need to be delivered to the nodes for effectively finalising the contract policy. The registration process includes metrics such as supplier details, chain-code ID, and random values to maintain the transaction process. The functionality of transactions could be validated through the authority policy within the nodes and enrolled into the blockchain network.

The mathematical representation of the Blockchain-enabled Vertical Aggregation Model for Drug Traceability System illustrates the Aggregation function, including the encryption and decryption processes. The nodes in the blockchain network demonstrate a stakeholder in the pharmaceutical supply chain, where every node has a data point that must be vertically aggregated to ensure effective data sharing. The procedure for vertical aggregation is computed in Eq. (1).

1 $$\:A{D}_{i}=Fn\left(D{p}_{i1},\:D{p}_{i2},\dots\:.,D{p}_{in}\right)\:\:\:\:\:\:\:\:\:\:\:\:$$

Where N is the number of nodes, $\:D{p}_{i}$ demonstrates Data points at every node, $\:A{D}_{i}$ is the Aggregated data at every node, $\:Fn$ denotes the Aggregation function, $\:En$ illustrates the Encryption function, $\:D{p}_{ij}$ is the Data Point j at node $\:i$. The secured data sharing has every node could encrypt the data points with a public-key encryption in Eq. (2).

2 $$\:En\left(D{e}_{ij}\right)=E{n}_{pk}D{e}_{ij}\:\:\:\:\:\:\:\:\:\:$$

Where pk denotes the public key, $\:De$ denotes the Decryption function, the aggregated data at every node is computed in Eq. (3) through encrypted data points.

3 $$\:A{D}_{i}=Fn\left(En\left(D{e}_{i1}\right),\:En\left(D{e}_{i2}\right),\:\dots\:.,En\left(D{e}_{in}\right)\right)\:\:\:\:\:\:\:\:\:$$

The Blockchain-enabled Aggregation Function is computed in Eq. (4).

4 $$\:AF=\sum\:_{i=1}^{n}{w}_{i}D{e}_{sk}\left(En\left(D{e}_{i}\right)\right)\:\:\:\:\:\:\:$$

Where $\:{w}_{i}\:$is assigned weight of every data point, $\:sk$ demonstrates the private key value.

The registration procedure has been enabled through the specific number of peer nodes from the endorsement process, as the output is encrypted and saved with the signatures of cryptographic procedures within the peer nodes. The output value is known as the endorsement. It contains the values of the necessary data, called metadata, which includes the ID and the signature of the user, along with the response, in the saved transaction policy. The supplier node will be helpful in gathering endorsements while it completes the policy and the transaction is committed, as the entire security policy is completed within the specified period. Whenever the transaction procedure is validated, it must be delivered to perform the ordering process. The endorsement state of the proposed work involves the manufacturer node gathering responses from the peer nodes. The ordering process involves broadcasting the transaction payload, gathering metadata, and developing a set of agreements. The ordering process employs a consensus methodology to determine and establish the execution order for every completed transaction in the system. Additionally, the transaction has the hash value of blocks with the endorsed transactions, enabling the services of ordering. The consignment state within the blocks is used to enhance the entire throughput and deliver the transaction-based cycle in the network, which is known as the execution state.

The local model $\:\left(lm\right)$ is computed in Eq. (5).

5 $$\:lm=\left\{{lm}_{1}^{tr},{lm}_{2}^{tr},\dots\:,{lm}_{\beta\:}^{tr}\:\right\}\:\:\:\:\:\:\:\:\:\:\:\:\:$$

Where $\:tr$ denotes the local training round, $\:\beta\:$ is the node id. The global model $\:\left(gm\right)$ is computed in Eq. (6).

6 $$\:gm=\left\{{gm}_{1}^{gr},\:{gm}_{2}^{gr},\dots\:.,{gm}_{\gamma\:}^{gr}\right\}\:\:\:\:\:\:\:\:\:\:\:\:\:$$

Where $\:gr$ is the global round value, $\:\gamma\:$ denotes the ID of the aggregation node, it demonstrates the comprehensive structure of the gathered data into the network by combining the data characteristics of every participant. The Aggregation node $\:\left(Ag\right)$ is computed in Eq. (7).

7 $$\:Ag=\left\{A{g}_{1},A{g}_{2},\dots\:.,A{g}_{\beta\:}\right\}\:\:\:\:\:\:\:\:\:\:\:\:\:$$

The Local training node (Lo) has the responsibility for training the local model, which is then delivered to the aggregation nodes of the global model, as computed in Eq. (8).

8 $$\:Lo=\left\{L{o}_{1},L{o}_{2},\dots\:,L{o}_{\gamma\:}\right\}\:\:\:\:\:\:\:\:\:\:\:$$

A blockchain node in the network provides a ledger through an aggregation mechanism involving a large number of nodes to enhance blockchain security. The proposed technique implements the aggregation functionality by initiating random successive blocks, which are sampled from several blockchain techniques, while the total blocks of every blockchain process have been initialised through Eq. (9).

9 $$\:Bc=\left\{B{c}_{1},B{c}_{2},\:\dots\:\dots\:.,\:B{c}_{\gamma\:}\right\}\:\:\:\:\:\:\:\:\:$$

The Secured Vertical Aggregation (SVA) provides robust aggregation of local models into the user, which are enrolled in specific peers for processing the chain code. This is achieved by delivering private data into a transient field to save updates to the local models. The policy determines which peers communicate the private information, as the count is initialised to zero, preventing the distribution process, while the maximum count is used to distribute the private information to minimise redundancy. The main concept is that the authorized peers have to be permitted for the process of reading and writing while the endorsement peers of the authorized executors do the partition of the local updates whenever the attributes and objects have been involved to perform the local model updates from the gathered information. The separate private data collections have to do the vertical partitioning of training data while the peer gathers private information of the training channel from the entire private data connections that every peer has to access the distributed models for validating the distributions to aggregate the process.

The proposed Secured Vertical Aggregation algorithm assigns the Euclidean weights for completing the local model updates which executes each peer to hold the distributions, the cosine score is used to compute the cosine weights in each sparse distribution by assigning the outlier score to zero and the updated coordinate weight to perform the average weight while the peer aggregates the channel distribution accessible to the remaining peers in the channel. The final global model updates have achieved the robust, secured aggregation from the present participants, the partitioned model to remove the biased updates.

**Fig. 4:**
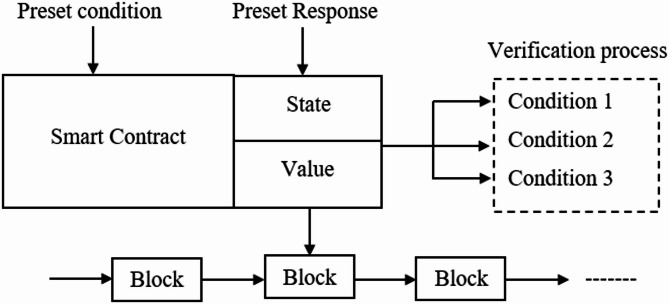
Smart Contract.

The smart contracts have the chain-code to ensure the successful execution of the entire rules to be included in the decentralized trust, the secured prediction functionality is used to perform the secured query processing of the global model for protecting the querying information. The real-time data is gathered through several sensors to maintain the services from the produced results through aggregated nodes. The preset conditions with the responses have been used for process automation to confirm the transaction integrity. The leveraging of the blockchain model with smart contracts ensures a secure way for executing contracts to adhere to the rules. The deployment process ensures transparency whenever the preset conditions are satisfied; the agreement is implemented automatically as the outcome is saved into the Blockchain. The power utilisation in real-time applications on local devices is reduced, allowing for data processing with minimal power consumption. The required energy and power utilisation are demonstrated through offloading data for further processing in cloud computing, as illustrated in Fig. 4.

In the proposed framework, the large training data is segregated into several parts, which require maximum power utilisation, time, and cost. The inference channel is enrolled into a proposal by endorsing peers for invoking the chain-code, the querying peer gathers the prediction values from the private information to produce the inference result, and the proposed technique produces secure prediction into the query model which is demonstrated in the Algorithm.

**Table:** Algorithm. Secured vertical aggregation (SVA).

Input: $\:\alpha\:$: number of participants, $\:g{m}^{t}$: Present global model, $\:v{p}_{m}$: Vertically partitioned model Output: $\:g{m}^{t+1}$: Produced aggregated model Begin Procedure $\:Coo{r}_{wt}\left(v{p}_{m},{\text{cos}}_{{wt}},len,\right)$ $\:wt\:=\:0$; $\:eu{c}_{wt}=0;$ $\:{stacked}_{{vp}_{m}}=stack\left(\left[m\in\:v{p}_{m}\right]\right);$ $\:sorte{d}_{v{p}_{m}}=sort\left({stacked}_{{vp}_{m}}\right);\:$ $\:{min}_{val}=sorte{d}_{v{p}_{m}}\left[\alpha\:\right];$ $\:{max}_{val}=sorte{d}_{v{p}_{m}}\left[len-\alpha\:\right];$ for every $\:i$ do for every $\:k\in\:n$ do if $\:(m\left[k\right]>{min}_{val}\left[k\right])\:$then if $\:(m\left[k\right]<{max}_{val}\left[k\right])\:$then $\:eu{c}_{wt}\left[i\right]\left[k\right]=1$; end if end if $\:wt\left[i\right]\left[k\right]=\:eu{c}_{wt}\left[i\right]\left[k\right]*{\text{c}\text{o}\text{s}}_{wt}\left[i\right];$ end for return $\:wt;$ End Procedure Begin Procedure $\:{\text{c}\text{o}\text{s}}_{wt}\left(v{p}_{m},wt\right)$ $\:{\text{c}\text{o}\text{s}}_{wt}=0;$ for every $\:i\:$do $\:{\text{c}\text{o}\text{s}}_{wt}\left[i\right]=\text{cos}\left(m,wt\right);$ end for return $\:{\text{c}\text{o}\text{s}}_{wt};$ End Procedure Begin Procedure $\:lates{t}_{aggregation}$ $\:len=len\left(v{p}_{m}\right);\:$ $\:{\text{c}\text{o}\text{s}}_{wt}=\:{\text{c}\text{o}\text{s}}_{wt}\left(v{p}_{m},wt\right);$ $\:wt\:=\:Coo{r}_{wt}\left(v{p}_{m},{\text{cos}}_{{wt}},len,\right);$ for every $\:k\in\:n$ do $\:\varDelta\:{wt}_{k}^{t+1}=\frac{\sum\:_{i=0}^{len}v{p}_{m}\left[i\right]\left[k\right]*wt\left[i\right]\left[k\right]}{wt\left[i\right]\left[k\right]};$ end for $\:w{t}^{t+1}=w{t}^{t}-\:\varDelta\:w{t}^{t+1};$ return $\:w{t}^{t+1};$ End Procedure	

##  Performance evaluation

The performance of the proposed Blockchain-enabled Secured Vertical Aggregation Algorithm for Drug Traceability Framework is evaluated by conducting repeated experiments in a simulated environment with the performance metrics like the Transaction Processing Time as the time required for processing a transaction which includes the time required to perform the aggregation process, the transactions per second which could be processed the total transactions. The Intel Core i7 processor is used to complete the performance evaluation with 16 GB RAM, 512 GB SSD storage, the network bandwidth of 100.0 Mbps, the Ubuntu 20.04.6 LTS operating system is involved at the Docker version of 24.0.7, the highest transaction per block is 500, the pool capacity for transaction is 20,000 with the 1s block interval and 2 MB of block size. The experimental analysis is performed into the main parts of communication cost, time, and blockchain performance analysis that the blockchain analysis performs the processing abilities and effectiveness whenever managing huge transactions, the time performance and the communication cost has the solution of data transmission overhead within the transaction process. The throughput is the total number of transactions processed within the unit time; the latency is the delay within the transaction by confirming the successful transmission. The Hyperledger fabric platform is used into the Blockchain transaction with the transmission rate of 1000 transactions per second from the Blockchain nodes. The algorithm demonstrated excellent scalability, with the ability to handle an increasing number of transactions and nodes without significant degradation in performance. The proposed BSVA technique is compared with the relevant techniques of BBTCD^18^, SecureMeds^19^, NFT-IoT Pharma Chain^25^, PHTrack^4^.

**Fig. 5:**
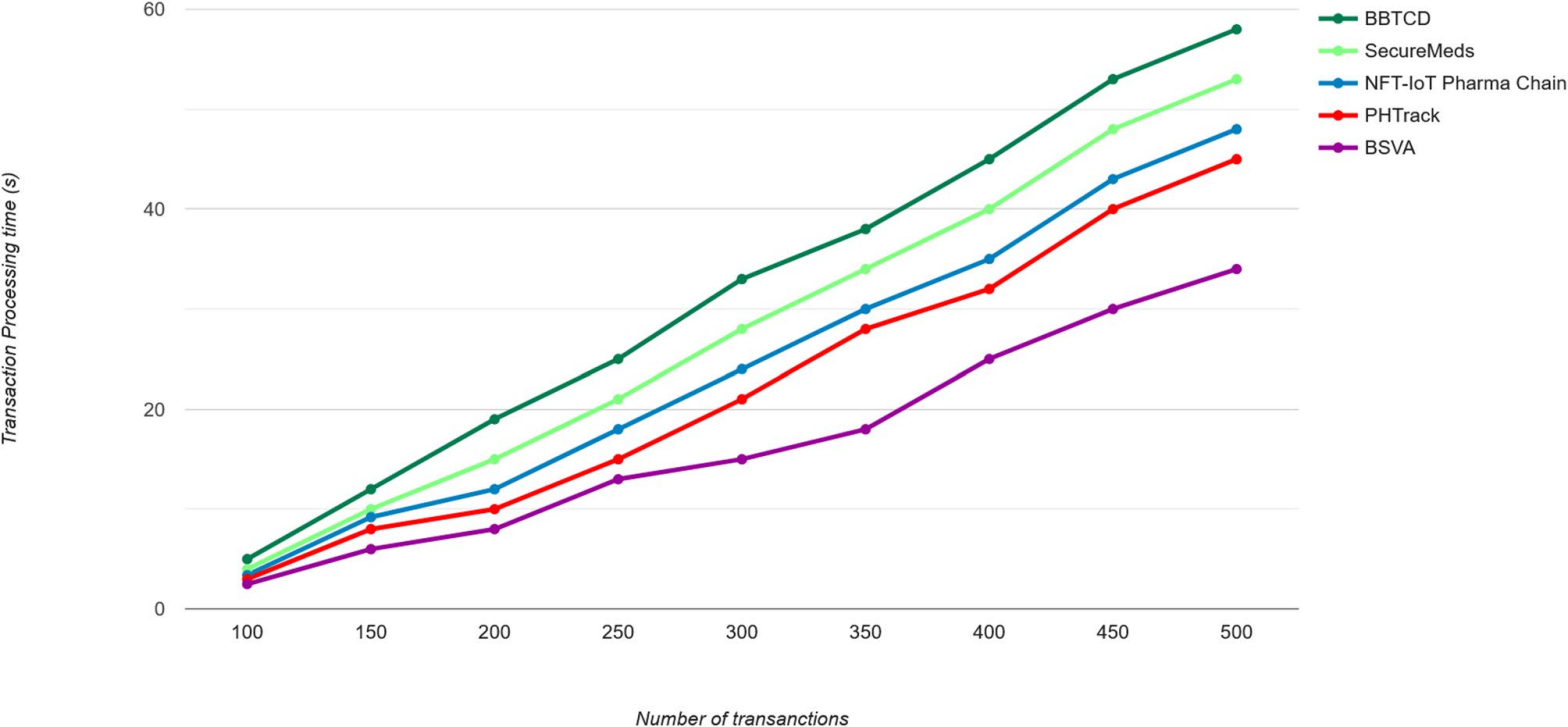
Transaction processing time.

The proposed Blockchain-enabled Secured Vertical Aggregation Algorithm for Drug Traceability Framework demonstrated excellent performance in terms of transaction processing time, transactions per second, throughput, latency, and scalability. The algorithm’s ability to handle a large number of transactions and nodes makes it suitable for large-scale drug traceability frameworks. The relationship within transaction processing time with the total blockchain nodes that have been analysed of various transaction loads, the node coordination enabled transmission overload has the impact of minimal processing time difference up to the transaction volumes and the results are changed for the proposed technique compared to the relevant techniques due to the processing efficiency, the node scaling functionality is used to handle huge amount of authentication request in spite of any communication overload. The results proved that the proposed technique can successfully enhance the node scaling functionality in a large volume of IoT authentication-related scenarios under the present network configuration in Fig. 5.

**Fig. 6:**
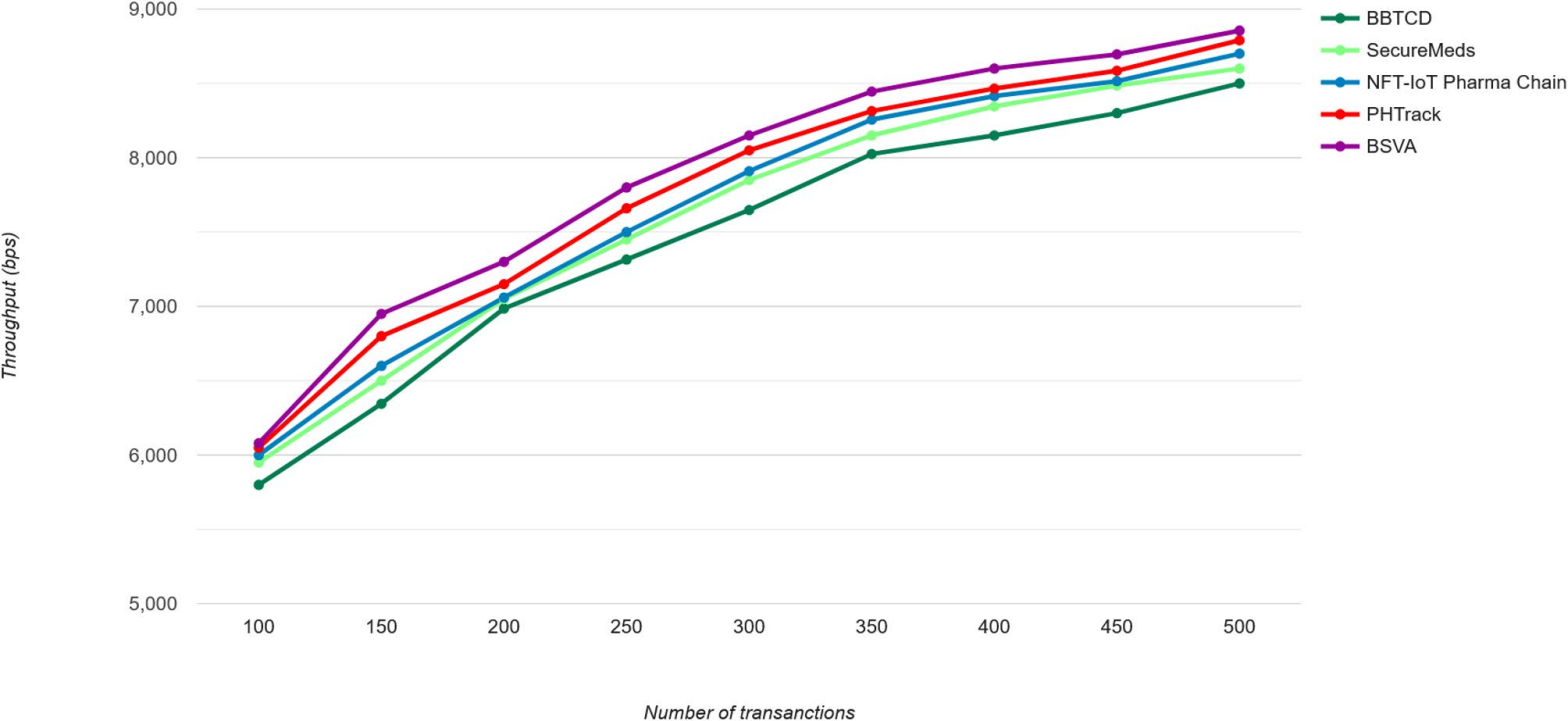
Throughput.

The proposed technique has improved system throughput as well as scalability advancements, the capability of the transaction processing is handled the system efficiently despite managing the authentication requests. While increasing the node size, the horizontal scaling approach has indicated the efficiency of the authentication process, the stabilized throughout into the maximum transaction volume managed through the expected processing capacity that is bounded with the consensus internal metrics and the block size, the transaction per second for the various techniques as the proposed work achieves a remarkable throughput of 250 transactions per second (TPS), surpassing existing techniques. The proposed model has produced the highest throughput from the aggregation model to establish the effective processing with the blockchain framework to provide efficient data management and the comparison of the proposed model with the relevant techniques is illustrated in Fig. 6.

**Fig. 7:**
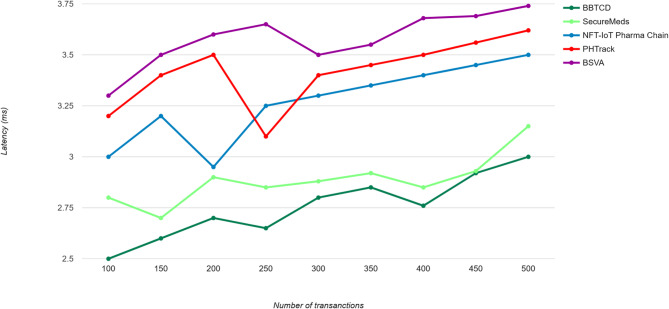
Latency.

The proposed model produces a reduced latency through the effective vertical aggregation algorithm to enable data aggregation with the utilization of blockchain technique for secured data storage and verification. The decentralized framework permits for distributed data processing with minimized latency to contribute the enhanced performance as the proposed model has the advantages of security and efficiency into the pharmaceutical industry. The capability of the proposed model is to afford real-time visibility for tracking the drug traceability process by minimizing counterfeiting to enhance the safety of the patient. Moreover, the proposed model provides a reasonable latency reduction compared with the relevant technique which is illustrated in Fig. 7.

**Fig. 8:**
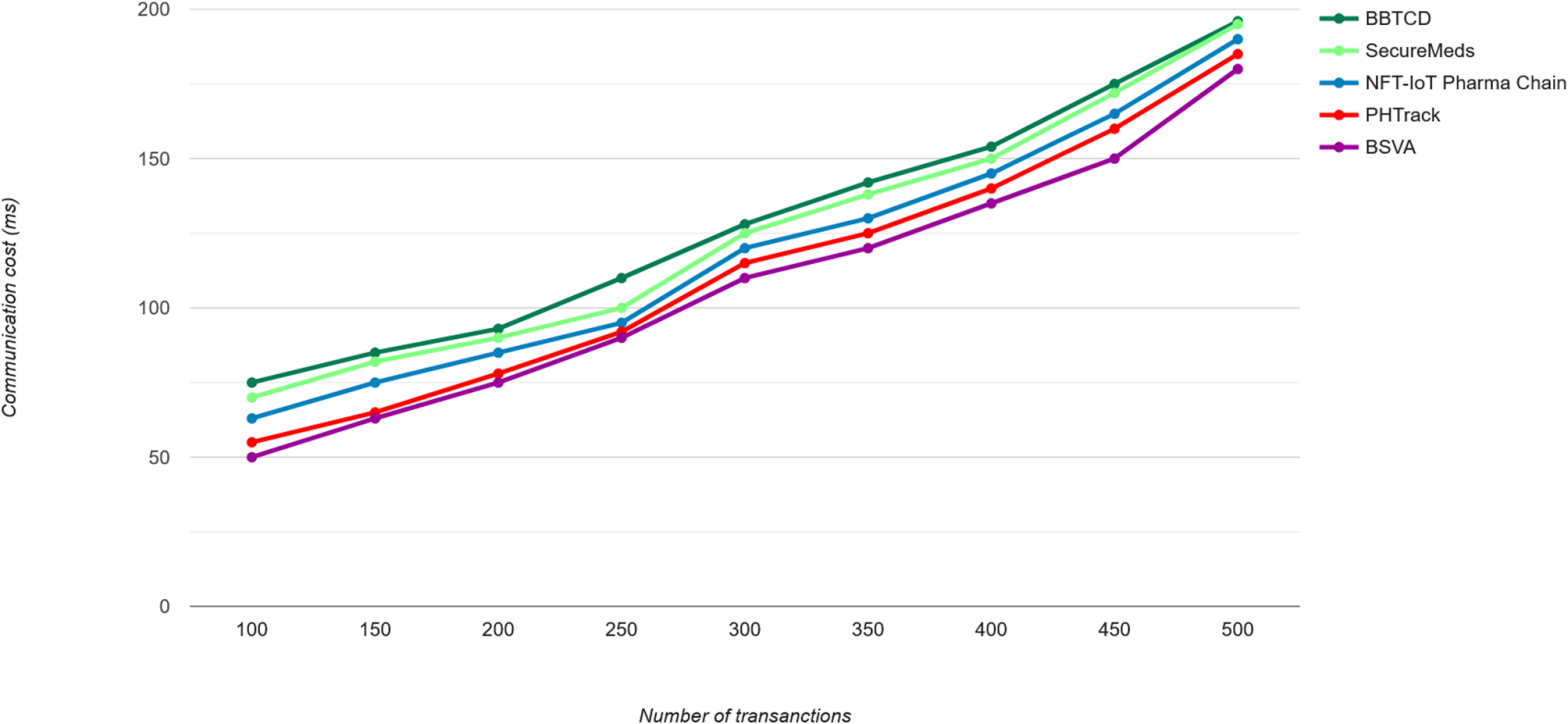
Communication cost.

The decentralized framework for the proposed model is used to permit the distributed data to be processed with minimized communication overload to contribute to improved performance. The minimized communication cost for the proposed model through the novel vertical aggregation algorithm to enable effective data aggregation process with the usage of blockchain technique and the comparison of the proposed technique with the relevant techniques is illustrated in Fig. 8.

The proposed BSVA algorithm has been deployed in a pharmaceutical supply chain to enhance traceability and transaction management. This is because distributors are responsible for transporting products to the companies, which can store them for distribution. The Retailers are involved into the Pharmacies and Hospitals to dispense medicines. The Regularity Bodies as the Government agencies to monitor the pharmaceutical industry. Table 1 demonstrates the benefits of utilizing Blockchain-enabled technique in the supply chain model to include the improved traceability, efficiency and regularity compliances. The Experimental result proved that the proposed model has the improved performance than the relevant techniques.

**Table 1:** Computational complexity comparison.

Parameters	BBTCD^18^	SecureMeds ^19^	NFT-IoT Pharma Chain^25^	PHTrack^4^	Proposed BSVA
Traceability	Low	Low	Medium	High	High
Security	Low	Medium	High	Low	High
Data integrity	Medium	Low	Medium	High	High
Interoperability	Low	Medium	High	Medium	High
Scalability	Low	Medium	Low	High	High
Energy Efficiency	Low	Low	Medium	Low	Medium
Latency	Medium	Low	High	Medium	Low

The computational complexity of the proposed model is the main component for considering the performance analysis with the related techniques, which leverage the blockchain model to ensure a secured data aggregation model to efficiently manage data. The proposed model maintains a balance between scalability and efficiency, its linear computational complexity is suitable for large-scale applications to ensure secured data aggregation and its vertical aggregation element increases the efficiency by minimizing the quality of data required to be transmitted and the comparison of computational complexity is demonstrated in Table 2.

**Table 2:** Computational complexity comparison.

Method	Computational complexity
BBTCD^18^	$\:O\left({n}^{2}\right)$
SecureMeds^19^	$\:O\left({n}^{3}\right)$
NFT-IoT Pharma Chain^25^	$\:O\left({n}^{2}\text{log}n\right)$
PHTrack^4^	$\:O\left(n\right)$
Proposed BSVA	$\:O\left(n\text{log}n\right)$

## Conclusion

The proposed Blockchain-enabled Secured Vertical Aggregation Algorithm provides a robust solution for authentication-related issues of pharmaceutical products through supply chain management. The centralised authority utilises the Hyperledger model to leverage the blockchain model, ensuring privacy and scalability while reducing response time for storing and sharing blockchain transactions. The utilisation of Centralised authority, Peers, Executors, and Orderer techniques ensures secure data sharing to reduce the tampering and counterfeiting process through the blockchain framework. The proposed BSVA algorithm is used to achieve the secured, robust and traceability through several key steps. Initially, the proposed algorithm uses a decentralized network for validating the transactions by ensuring the tamper-proof. The blockchain model provides a secure and robust framework for data management, enabling the creation of immutable records. The proposed algorithm has the advanced encryption model for protecting sensitive data. The smart contract is used to automate the verification process by ensuring the consistent and accurate data. The Vertical Aggregation functionality permits the effective analysis of data from the sources to provide the complete view of the supply chain. The successful implementation of the proposed algorithm has been utilised in the pharmaceutical industry to regulate both consumers and manufacturers by enabling real-time tracking, ensuring the overall efficiency of the supply chain management system. In the future, emerging technologies like AI and IoT will enhance the proposed model’s capability to conduct pilot studies and collaborate with industry stakeholders for validating the efficiency of the proposed solution in real-time environments.

## Data Availability

The datasets generated and/or analyzed during the current study are available from the corresponding author on reasonable request.
